# Hyperbaric Oxygen Therapy Versus Intravenous Thrombolysis in the Treatment of Central Retinal Artery Occlusion: A Systematic Review and Meta-Analysis

**DOI:** 10.3390/jcm15072628

**Published:** 2026-03-30

**Authors:** Anas Bakdalieh, Dawood Siddiqui, Ding-Geng Chen, Minzhong Yu

**Affiliations:** 1 College of Medicine, Northeast Ohio Medical University, Rootstown, OH 44272, USA; abakdalieh@neomed.edu (A.B.); dsiddiqui@neomed.edu (D.S.); 2 College of Health Solutions, Arizona State University, Phoenix, AZ 85004, USA; ding-geng.chen@asu.edu; 3 Department of Statistics, University of Pretoria, Pretoria 0002, South Africa; 4 Department of Ophthalmology and Visual Sciences, University Hospitals Eye Institute, Case Western Reserve University, Cleveland, OH 44106, USA; 5 Cole Eye Institute, Cleveland Clinic Foundation, Cleveland, OH 44106, USA; 6 Department of Ophthalmology, Cleveland Clinic Lerner College of Medicine of Case Western Reserve University, Cleveland, OH 44195, USA

**Keywords:** central retinal artery occlusion, hyperbaric oxygen therapy, intravenous thrombolysis, best-corrected visual acuity, systematic review and meta-analysis

## Abstract

**Background:** Central retinal artery occlusion (CRAO) causes sudden, often profound monocular vision loss. We compared the efficacy and safety of hyperbaric oxygen therapy (HBOT) versus intravenous thrombolysis (IVT) using a systematic review and meta-analysis. **Methods:** Following PRISMA 2020 guidance, we searched PubMed, Cochrane Library, Web of Science, and VHL from inception to 27 May 2025, screened records in duplicate, and synthesized visual outcomes (best-corrected visual acuity [BCVA], logMAR), onset-to-treatment time, and adverse events using random-effects models. **Results:** Twenty-five observational studies (781 patients; 557 HBOT eyes, 225 IVT eyes) met inclusion. Both HBOT and IVT were associated with significant improvements in BCVA (HBOT: MD −0.57 logMAR; IVT: MD −0.53 logMAR). Clinically meaningful improvement occurred in 45.8% after HBOT and 42.0% after IVT. Adverse event rates were similar (HBOT 11.3%; IVT 10.2%) but differed in type (ear barotrauma with HBOT; hemorrhagic events with IVT). **Conclusions:** HBOT and IVT both improve visual outcomes in CRAO. Differences in adverse event profiles and substantial heterogeneity among IVT studies underscore the need for adequately powered comparative trials and standardized treatment pathways. Registration: Not prospectively registered; PRISMA 2020 checklist is provided.

## 1. Introduction

Clinical guidance recognizes acute CRAO as an ophthalmic form of acute ischemic stroke requiring urgent evaluation under stroke protocols. Recent AHA statements and AAO Preferred Practice Patterns emphasize immediate triage, arteritis screening in older adults, and coordinated stroke-system management. Within this context, IVT may be considered in carefully selected patients within 4.5 h, whereas HBOT availability and timing vary by center [1,2,3].

Ongoing controversies relate to (i) candidate selection and contraindications for systemic thrombolysis, including hemorrhagic risk, and (ii) feasibility and timing of HBOT, which often occurs outside thrombolysis windows. These pathway differences complicate cross-modality comparisons and motivate a cautious, uncertainty-aware synthesis rather than practice-changing claims [1,2,3,4,5].

Because clinicians frequently must choose between IVT and HBOT based on onset-to-treatment time, contraindications, and local access, we synthesize within-modality visual outcomes and safety profiles to inform pragmatic decision-making when randomized head-to-head trials are unavailable. We therefore moderate novelty to ‘comprehensive observational synthesis’ and frame conclusions as hypothesis-generating.

Central retinal artery occlusion (CRAO) is a severe ophthalmic disorder caused by blockage of the central retinal artery, often from an embolism, leading to sudden vision loss [6]. It affects about 1 in 100,000 people annually, mainly men over 60, and has a poor prognosis, with only ~17.7% recovering without treatment [7].

The natural history of CRAO is poor, with spontaneous visual recovery occurring in only a minority of patients despite the acute onset of symptoms. Prognosis is strongly influenced by several clinical modifiers, including baseline visual acuity, ischemic duration, embolus composition, or retrobulbar spot-sign status, and imaging features such as OCT indicators of inner retinal ischemia. Arteritic CRAO represents a pathophysiologically distinct condition with substantially worse outcomes and is typically excluded from interventional studies, including those synthesized in this review. Beyond visual acuity metrics, CRAO can significantly impair reading, driving eligibility, and daily functioning, underscoring the clinical importance of even modest improvements in logMAR outcomes. Incorporating these prognostic and functional parameters strengthens the rationale for synthesizing treatment-specific visual outcomes across HBOT and IVT.

These epidemiologic and prognostic features, including low spontaneous recovery, strong time dependence, and modifier effects, provide the context for interpreting our pooled estimates and reinforce our caution about indirect, cross-modality comparisons.

Although there is no current guideline-approved treatment for CRAO, several non-invasive and invasive treatments have shown some efficacy. Some of the non-invasive approaches include ocular massage, antiplatelet therapy, and hyperbaric oxygen therapy (HBOT) [8]. Some invasive approaches to treating CRAO include anterior chamber paracentesis, intra-arterial thrombolysis, and intravenous thrombolysis (IVT) [9].

HBOT increases the partial pressure of oxygen, and subsequently its concentration, in the retina. The retina has one of the highest oxygen consumption rates in the body, which supports its potential as a therapeutic strategy [10]. Common side effects caused by HBOT include middle ear barotrauma and claustrophobia due to the design of the chamber [11]. Another common treatment for CRAO is IVT, which lyses the embolism in the central retinal artery, thereby improving perfusion [12]. The efficacy of IVT involves the specific size and location of the clot, which varies from patient to patient. Within 4.5 h of the onset of symptoms, patients receive intravenous alteplase at a standard dose of 0.9 mg/kg, with 10% bolus administered within the first minute, and the remainder over the course of an hour [13]. The side effects pertaining to IVT include excessive bleeding and intracranial hemorrhage [14].

Although HBOT and IVT have been studied separately in CRAO, this work provides a comprehensive observational systematic review (SR) from all sources of published studies with a meta-analysis (MA) to synthesize all evidence to compare visual outcomes and adverse events for both treatments so a statistically more powerful conclusion can be made for evidence-based clinical recommendation and practice.

Here, we aim to fill this knowledge gap to present a comprehensive observational synthesis directly comparing HBOT and IVT for CRAO using systematic review and meta-analysis to inform evidence-based clinical decision-making.

Our prespecified primary outcome was the change in best-corrected visual acuity (BCVA) in logMAR from pre- to post-treatment. We conducted modality-specific syntheses of observational cohorts receiving hyperbaric oxygen therapy (HBOT) or intravenous thrombolysis (IVT) and provided indirect, qualitative cross-modality interpretation given the lack of head-to-head randomized trials. The intended clinical implication is pragmatic: to contextualize visual outcomes and adverse event profiles by modality to aid time-sensitive decision-making across diverse care pathways, while avoiding claims of definitive comparative efficacy.

### 1.1. Pathophysiology and Ischemic Tolerance of the Inner Retina

The central retinal artery is a terminal artery; sudden occlusion abruptly compromises inner retinal perfusion and oxygen delivery. Metabolic demand in ganglion cells and inner plexiform layers is among the highest in the CNS, leading to rapid ionic pump failure, cytotoxic edema, and visual loss within minutes. Experimental and clinical observations suggest a narrow, time-dependent window of potential reversibility, consistent with the clinical imperative for ultra-early reperfusion or oxygen augmentation. These fundamentals frame the rationale for evaluating HBOT (which increases dissolved oxygen content and augments choroidal-to-retinal diffusion) and IVT (which restores arterial patency by fibrinolysis) [6,7,8,10,12,13,14].

### 1.2. Epidemiology and Natural History

CRAO is uncommon, but vision-threatening, with annual incidence estimates roughly on the order of 1 per 100,000 and strong age and vascular risk enrichment. Spontaneous visual recovery is generally poor: most cohorts report only a minority achieving clinically meaningful gains without targeted therapy, and baseline severity and ischemic duration are dominant determinants of prognosis. Functional repercussions extend well beyond letter scores, including loss of driving eligibility, impaired reading, and reduced independence, underscoring the clinical significance of even modest logMAR improvements. Prognosis is additionally shaped by clinical modifiers, such as arteritic versus non-arteritic etiology, embolus composition/“spot-sign” status, baseline BCVA, and OCT indicators of inner retinal ischemia, which also influence responsiveness to time-sensitive interventions. This natural-history context motivates our modality-specific synthesis focused on patient-centered BCVA change, acknowledging that therapeutic windows and access pathways (stroke-team IVT vs. referral-dependent HBOT) are likely to modulate real-world effectiveness.

### 1.3. Mechanistic Rationale for HBOT Versus IVT

HBOT elevates ambient pressure (typically 2.0–2.8 ATA) while administering nearly 100% oxygen, substantially increasing plasma-dissolved O_2_ and steepening the oxygen diffusion gradient from the choroid to the hypoxic inner retina. Beyond oxygen delivery, HBOT may transiently reduce edema and modulate inflammatory cascades—mechanisms that plausibly preserve tissue at risk even when arterial patency is not immediately restored. In contrast, IVT activates plasmin via recombinant tissue plasminogen activator (rtPA), promoting fibrin degradation and potential recanalization of the occluded central retinal artery. The benefit of IVT is highly time-sensitive and depends on thrombus composition and burden; risks are dominated by systemic and intracranial hemorrhage [10,12,13,14].

### 1.4. Clinical Context and Rationale for a Comparative Synthesis

Despite growing interest in implementing ‘stroke-like’ hyperacute pathways for CRAO, practice remains heterogeneous. Some centers prioritize IVT under existing stroke protocols within 4.5 h, whereas others refer to hyperbaric centers and initiate HBOT when feasible, sometimes beyond conventional thrombolytic windows. Given the absence of guideline-endorsed therapy, a rigorous synthesis of visual outcomes and safety across HBOT and IVT is needed to support pragmatic, context-dependent decision-making [8,12,13,15].

### 1.5. Clinical Guidance and Care Pathways

Acute CRAO is recognized as an ocular form of acute ischemic stroke that warrants immediate triage to an emergency department under stroke protocols. Recent statements and Preferred Practice Patterns emphasize urgent evaluation (including arteritis screening in older adults), rapid confirmation of diagnosis, and coordinated stroke-system management. In this context, IVT within 4.5 h may be considered for carefully selected patients, while hyperbaric oxygen therapy (HBOT) is variably available and often initiated later depending on local resources [1,2,3].

### 1.6. Rationale for a Comparative Synthesis

Although prior reviews have evaluated HBOT or IVT separately in CRAO, clinicians frequently must choose between these modalities based on time from onset, contraindications, and access. We therefore synthesized within-modality outcomes and described adverse event profiles to inform pragmatic decision-making when randomized, head-to-head trials are unavailable.

### 1.7. Novelty and Scope

This review collates observational evidence across both modalities and standardizes visual outcomes (logMAR) to enable modality-specific meta-analyses with qualitative cross-modality interpretation. We do not claim definitive comparative efficacy and, as such, frame conclusions as hypothesis-generating, given the indirect nature of comparisons.

## 2. Methods

Protocol and registration rationale: We drafted an internal protocol (PICOS, data items, and primary/secondary outcomes) before screening, but did not prospectively register it (e.g., PROSPERO) due to project timelines and the retrospective synthesis of already published data. To mitigate selective reporting, we prespecified the primary endpoint (change in BCVA, logMAR), archived search strings, and provided the PRISMA 2020 checklist in the Supplementary Materials [16].

Heterogeneity strategy: Fewer than ten studies contributed to most quantitative syntheses, limiting power for meta-regression or meaningful subgroup analyses. We therefore prespecified random-effects models, quantified heterogeneity with I^2^, and summarized clinically important sources (onset-to-treatment time, baseline acuity, embolus characteristics, and protocol differences) narratively, reserving pooled estimates for interpretable cases.

### 2.1. Protocol, Registration, and Reporting Standards

**Figure 1:**
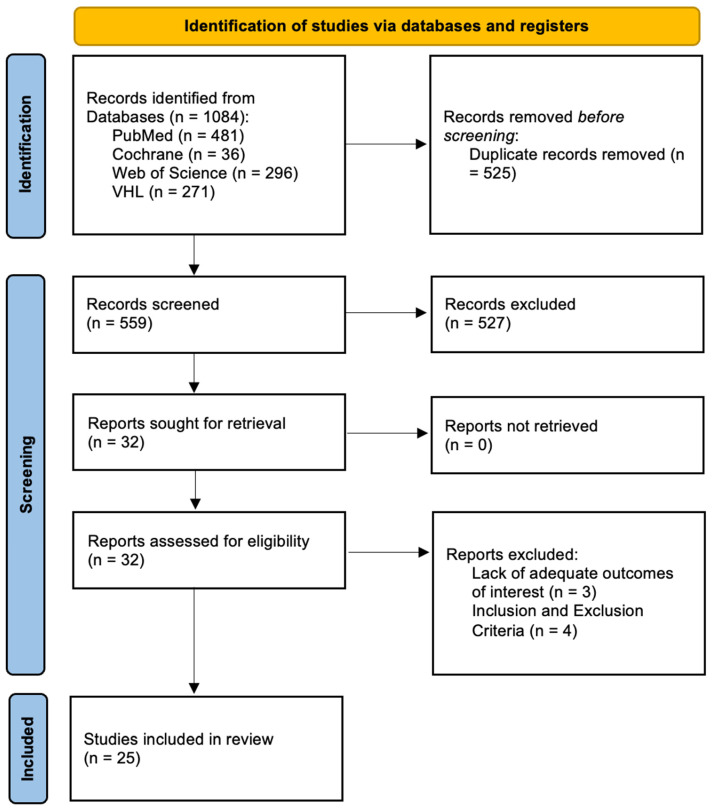
PRISMA flow diagram.

This review was conducted and reported in accordance with the PRISMA 2020 statement (Preferred Reporting Items for Systematic Reviews and Meta-Analyses). A completed PRISMA 2020 checklist is provided in Supplementary File S1, and the PRISMA 2020 flow diagram is presented as Figure 1. The review protocol was not prospectively registered (e.g., PROSPERO), consistent with PRISMA Item #24; no protocol amendments occurred after the review commenced.

### 2.2. Eligibility Criteria (PICOS)

Population: Human patients with central retinal artery occlusion (CRAO), non-arteritic unless otherwise specified.

Interventions: Hyperbaric oxygen therapy (HBOT) or IVT (IVT).

Comparators: Pre- versus post-treatment comparisons within arm; where available, between-treatment comparisons across studies.

Outcomes: Primary—change in BCVA (logMAR). Secondary—proportion with clinically meaningful BCVA improvement (≥0.3 logMAR), onset-to-treatment time, and adverse events.

Study design: Observational studies (prospective or retrospective) reporting relevant outcomes; case reports, reviews, editorials, and animal studies were excluded.

### 2.3. Information Sources and Search Strategy

We systematically searched PubMed, Cochrane Library, Web of Science, and VHL from inception through 27 May 2025, without language or date restrictions. Search strategies used controlled vocabulary and keywords for CRAO, hyperbaric oxygen, thrombolysis, tissue plasminogen activator, and alteplase; full strings are provided in Supplementary Table S1. Records were imported to Rayyan (QCRI) for de-duplication and screening.

### 2.4. Study Selection

Two reviewers (A.B., D.S.) independently screened titles/abstracts and assessed full texts against the eligibility criteria. Disagreements were resolved by discussion or consultation with a senior author (M.Y.). The study selection process is summarized in the PRISMA 2020 flow diagram (Figure 1).

### 2.5. Data Collection and Data Items

Using a piloted extraction form, two reviewers independently extracted study characteristics (author, year, country, design, setting), patient demographics (age, sex), treatment details (HBOT protocol; IVT agent/dose and timing), and outcomes (pre/post BCVA in logMAR, SDs where available; onset-to-treatment time; proportion achieving ≥0.3 logMAR improvement; adverse events). Any discrepancies were resolved by consensus or third-party adjudication (M.Y.).

### 2.6. Risk of Bias in Individual Studies

Risk of bias was assessed with the Newcastle–Ottawa Scale (NOS) for cohort studies. Studies were graded as Unsatisfactory, Satisfactory, Good, or Very Good according to NOS criteria; details are reported in Supplementary Table S5.

### 2.7. Effect Measures and Synthesis Methods

For continuous outcomes (BCVA, logMAR), mean differences (MD) with 95% confidence intervals (CIs) were calculated. For proportions (rate of clinically meaningful BCVA improvement; adverse events), we synthesized descriptive pooled rates. Random-effects models were used to account for between-study heterogeneity; heterogeneity was quantified using I^2^. Analyses were performed in RevMan 5.4.1.

The statistical approach followed standard methods for random-effects meta-analysis as detailed in [16]. Publication bias was not formally assessed because fewer than 10 studies contributed to each quantitative synthesis. No GRADE certainty assessment was undertaken.

### 2.8. Reporting Bias and Additional Analyses

No subgroup, sensitivity, or meta-regression analyses were prespecified. Where clinical or methodological heterogeneity precluded meta-analysis, results were summarized narratively.

### 2.9. Data Harmonization and Transformations

When studies reported visual acuity in non-logMAR formats (e.g., Snellen), we applied standard conversions to logMAR to maintain comparability across datasets. For studies providing medians and interquartile ranges, we followed established approximations to derive means and standard deviations when necessary, and we performed consistency checks against reported raw data. These steps were pre-planned to minimize transformation bias and were implemented only when essential to include a study in quantitative synthesis [16].

### 2.10. Statistical Model Justification

We prespecified random-effects models to allow for clinical and methodological heterogeneity across centers, imaging criteria, and treatment logistics. Effects were pooled using inverse-variance weighting, and between-study heterogeneity was quantified by I^2^. Where heterogeneity appeared substantial, we provided narrative synthesis to preserve interpretability rather than force a pooled estimate [16].

### 2.11. Handling of Multiplicity and Selective Reporting

To limit inflation of type I error from multiple outcomes, we designated the change in BCVA (logMAR) as the primary endpoint a priori and treated safety and onset-to-treatment time as key secondary endpoints. Because fewer than 10 studies contributed to any single meta-analysis, we did not perform funnel plot or Egger tests; instead, we qualitatively assessed small-study effects by inspecting precision and study sizes [16].

## 3. Results

**Figure 2:**
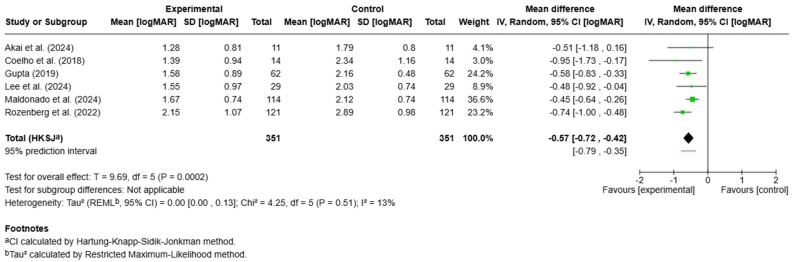
Forest plot of the mean difference in BCVA before and after HBOT treatment [17,18,19,20,21,22].

**Figure 3:**
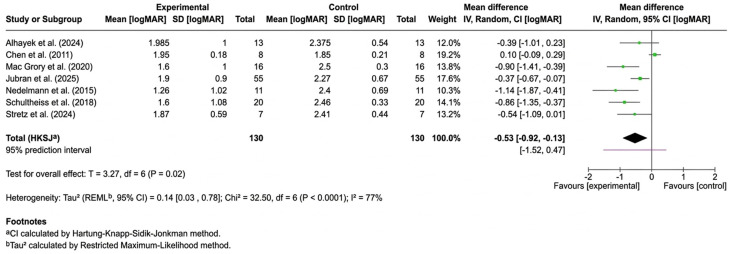
Forest plot of the mean difference in BCVA before and after IVT treatment [15,23,24,25,26,27,28].

Clarification of comparative framework: Figure 2 and Figure 3 present within-modality pre–post meta-analyses (HBOT and IVT, respectively); no direct head-to-head randomized comparisons were available. Cross-modality statements in this manuscript are therefore qualitative and indirect, intended to be hypothesis-generating rather than definitive.

### 3.1. Literature Search

Our initial literature search yielded 1084 studies, of which 525 duplicates were removed. Of the remaining 559 studies, 527 were excluded through title and abstract review. After performing full-text screening, 25 studies were included in this review [15,17,18,19,20,21,22,23,24,25,26,27,28,29,30,31,32,33,34,35,36,37,38,39,40]. Figure 1 depicts the PRISMA search strategy.

### 3.2. Study Characteristics

All 25 included studies were observational studies, with 19 being single-center and multicenter retrospective studies [17,18,20,21,22,23,25,26,28,30,31,32,33,34,35,36,37,38,39,40] and six being single and multicenter prospective studies [15,19,24,26,27,29]. A total of 781 patients (557 eyes undergoing HBOT and 225 eyes undergoing IVT) were included in these studies. All of the patients except one were treated for CRAO in one eye; one patient was treated in both eyes [17]. Sample sizes ranged from 7 to 121 patients [24,28]. The majority of the patients were male (62.6%, *n* = 755). The mean age, when reported, ranged from 60 years in the youngest group [40] to 74.3 years in the oldest group [33] (Table 1).

### 3.3. Visual Acuity Outcomes

In total, 21 studies [15,17,18,19,20,21,22,23,24,25,26,27,28,29,31,33,34,36,38,39,40] reported a difference in BCVA (logMAR) in patients before and after treatment. Among those, 13 studies [15,17,18,19,20,21,22,23,24,25,26,27,28] reported the mean BCVA and standard deviation (SD) (*n* = 481). BCVA 19 being single-center and multicenter retrospective studiessignificantly in patients in both the HBOT group (*p* = 0.0002; *n* = 351) and the IVT group (*p* = 0.02; *n* = 130). In the studies that investigated HBOT [17,18,19,20,21,22,26,29,30,31,32,33,34,35,36,37,38,39,40], the MD in BCVA before and after treatment was −0.57, 95% CI [−0.72, −0.42] (Figure 2). In the studies that investigated IVT [15,23,24,25,26,27,28], the MD in BCVA before and after treatment was −0.53, 95% CI [−0.92, −0.13] (Figure 3).

### 3.4. Rate of BCVA Improvement

There were 21 studies that reported on the proportion of patients who demonstrated a clinically significant improvement in visual acuity (*n* = 696). The pooled rate of BCVA improvement was 45.8% (*n* = 546) in the group of patients that underwent HBOT treatment [18,19,20,21,22,29,30,33,35,36,37,39,40]. For the patients who received IVT treatment, the pooled rate of BCVA improvement was 42.0% (*n* = 150) [15,24,26,27,31,32,34,38]. In most studies, a decrease in BCVA in logMAR of 0.3 or more was considered a significant improvement in visual acuity (Supplementary Table S2).

### 3.5. Onset-to-Treatment Time

The onset-to-treatment time was reported in 20 studies (*n* = 675) [17,20,21,22,25,33,34,38,39,40]. Of those, ten studies reported patients in the HBOT group (*n* = 457), and ten studies reported those in the IVT group (*n* = 218). The times reported varied widely among studies, with most patients being treated within 20 h of disease onset, while two studies reported patient treatment days after onset [20,40]. Onset-to-treatment time was notably higher in the HBOT group (Supplementary Table S3).

### 3.6. Adverse Events

Among all of the studies included, a total of 86 adverse events were reported in 18 studies (Supplementary Table S4). In the CRAO patients treated with HBOT, 63 patients experienced adverse events, with a pooled complication rate of 11.3% (*n* = 557) [17,18,19,21,22,29,30,33,35,37,39]. The most common adverse event was ear barotrauma (23, 36.5%) [18,21,22,29,30,33], followed by hypertension in 16 patients (25.4%) [29,30], neovascularization in 13 patients (20.6%) [33,35], a need for incisional myringotomies in nine patients (14.3%) [37], and hemotympanum in three patients (4.8%) [39]. Two patients experienced otalgia (3.2%) [19], two patients had anxiety (3.2%) [39], two experienced seizures and epistaxis (3.2%) [22], and two prematurely stopped the treatment (3.2%) [17]. Finally, one patient’s hearing loss worsened (1.6%) [17]. Notably, in the study that included two patients who experienced ear barotrauma and two patients who had seizures and epistaxis, the treatment of all four patients was prematurely stopped [22].

In the IVT group, 23 patients experienced adverse events, with a pooled complication rate of 10.2% (*n* = 225) [15,23,24,25,27,31,34]. Hemorrhages were the most common adverse event (7, 30.4%) [15,23,24,25,27,31], followed by ocular ischemic complications in six patients (26.1%) [34]. The hemorrhages consisted of different types, including four intracranial hemorrhages [23,24,31], one intracerebral hemorrhage [15], one minor systemic hemorrhage [25], and one hemorrhage from an abdominal aortic aneurysm [27]. Four patients experienced recurrent retinal artery occlusion (RAO) (17.4%) [25,27], one patient had a stroke (4.3%), one patient experienced angioedema (4.3%), one patient had a silent cerebral infarct (4.3%), and one patient experienced retinal neovascularization (4.3%) [24].

### 3.7. Risk of Bias

All 25 observational studies were assessed using the Newcastle–Ottawa Scale guidelines [41]. A total of 13 studies [17,18,19,21,26,28,29,30,33,36,37,39,40] (52%) received scores of 5 or 6 and were graded as “Satisfactory”, and 12 studies [15,20,22,23,24,25,27,31,32,34,35,38] (48%) received scores of 7 or 8 and were graded as “Good”, in accordance with the Newcastle–Ottawa criteria. One point was awarded for each of the following: representativeness of the exposed cohort; representativeness of the non-exposed cohort; ascertainment of exposure; demonstration that outcome of interest was not present at start of the study; assessment of outcome; adequate duration of follow-up; adequate follow-up of cohorts. One to two points were awarded for comparability of cohorts based on the design or analysis. The results of the Newcastle–Ottawa Scale are shown in Supplementary Table S5.

### 3.8. Qualitative Sources of Heterogeneity

Across IVT cohorts, heterogeneity likely reflected differences in onset-to-needle times, thrombus composition (e.g., calcific vs. cholesterol emboli), rtPA dosing nuances, and co-management within stroke pathways. HBOT cohorts varied by chamber availability, number and pressure of dives, and delays from symptom onset, which can lengthen when referral to hyperbaric centers is required. These practical differences contextualize the wider confidence intervals observed in IVT and the relatively tighter estimates among HBOT reports [13,20,22,26,28,40].

### 3.9. Clinical Meaningfulness of BCVA Changes

The proportion achieving ≥0.3 logMAR improvement provides a patient-centered yardstick: nearly half of HBOT-treated patients and ~40% of IVT-treated patients met this threshold across included cohorts, albeit with variable follow-up durations and baseline severities. Such changes translate into meaningful functional differences in daily activities, supporting the clinical relevance of both modalities when applied in appropriate windows and settings [15,18,19,20,21,22,24,26,27,28,29,30,31,33,35,36,38,39,40].

## 4. Discussion

Interpretation and clinical caution: Apparent similarity in average BCVA gains across modalities should be interpreted cautiously, given observational designs, indirect comparisons, and between-study heterogeneity, particularly among IVT cohorts. Confounding by indication, referral and access patterns (e.g., availability of hyperbaric centers), and onset-to-treatment timing likely influenced outcomes. Accordingly, our findings should be interpreted as hypothesis-generating rather than practice-changing, reflecting the constraints of indirect comparisons across heterogeneous observational data. Given these limitations, any apparent similarity in BCVA improvement between HBOT and IVT should be interpreted strictly as an indirect observation rather than evidence of true comparative efficacy.

Emerging evidence highlights that embolus composition—particularly calcific versus cholesterol or platelet-fibrin material—and retrobulbar “spot-sign” status strongly influence the likelihood of recanalization with IVT. Calcific emboli, which frequently generate a positive spot sign on B-scan ultrasonography, are generally resistant to fibrinolysis and rarely respond to rtPA-based thrombolysis. In contrast, fibrin-rich emboli are more amenable to enzymatic degradation. These mechanistic distinctions limit the biological plausibility of IVT as a universally effective reperfusion strategy across all CRAO phenotypes. As a result, the observed pre–post visual acuity improvements in IVT cohorts may partially reflect the spontaneous recovery expected in selected, time-favorable patients rather than consistent thrombus dissolution. Recognizing the heterogeneity of embolus composition underscores the need to interpret indirect cross-study comparisons cautiously and reinforces that HBOT, an oxygen-augmentation therapy without reperfusion intent, should not be construed as mechanistically equivalent to IVT or other reperfusion-directed approaches.

Additional sources of bias likely influenced observed outcomes. Confounding by indication is inherent in CRAO pathways, as candidates for IVT are typically selected based on stroke-team triage criteria, baseline acuity, comorbidities, and time from onset, whereas HBOT cohorts may reflect later presenters or those ineligible for thrombolysis. Referral and access patterns—including limited proximity to hyperbaric centers—introduce further systematic differences in onset-to-treatment time that may bias visual outcomes in favor of IVT or HBOT depending on local logistics. These structural and clinical factors reinforce that cross-modality interpretation must remain hypothesis-generating rather than comparative.

Contraindications and selection effects: IVT candidacy is constrained by standard alteplase exclusions (e.g., recent surgery, anticoagulant effect, uncontrolled hypertension), which may have reduced hemorrhagic events in observational cohorts via selection and timing. HBOT carries its own exclusions (e.g., untreated pneumothorax) and relative risks (e.g., middle ear barotrauma, oxygen-toxicity seizures, claustrophobia). These non-overlapping constraints shape the observed safety profiles [1,5].

Timing sensitivity: The therapeutic window differs materially between IVT (typically ≤4.5 h) and HBOT (often later due to referral and access). Emerging reports suggest that earlier HBOT may yield greater visual benefit, reinforcing streamlined ‘time is eye’ pathways [3,33,42,43].

This comparative synthesis indicates that both HBOT and IVT can yield clinically meaningful visual gains in CRAO, with broadly similar average magnitudes of BCVA improvement. However, precision and consistency were higher among HBOT studies, while IVT estimates were heterogeneous, likely reflecting variation in embolus composition (e.g., spot-sign-positive calcific emboli), time-to-treatment, dosing, and adjunctive care.

Choice of therapy must balance feasibility and safety. In practice, IVT is often available early under stroke pathways (≤4.5 h) but carries hemorrhagic risk, whereas HBOT access is limited to centers with chambers and may be initiated later, predisposing to ear-related barotrauma. These distinct adverse event profiles mirror the different mechanisms (systemic fibrinolysis vs. pressurization/oxygenation).

Our review has limitations: the evidence base is observational without randomized clinical trials with head-to-head comparisons; sample sizes are modest; and between-study heterogeneity—particularly for IVT—limits predictive validity. Future trials should directly compare HBOT versus IVT, standardize eligibility (including imaging-based embolus characterization), and incorporate patient-centered outcomes and safety monitoring.

### 4.1. Mechanistic Interpretation of Adverse Events

Ear barotrauma predominated with HBOT, consistent with pressure-related middle-ear injury risk; anxiety and claustrophobia occasionally necessitated early termination. In contrast, hemorrhagic events, ranging from minor systemic bleeds to intracranial hemorrhage, dominated the IVT risk spectrum, mirroring experience in ischemic stroke thrombolysis. These distinct safety signatures, alongside feasibility constraints, should shape individualized treatment selection [11,14,15,22,23,24,25,27,31,39].

Prostaglandin E1 (PGE1) has recently gained attention as a third mechanism-based therapeutic candidate for CRAO. Unlike IVT, which enzymatically lyses emboli, and unlike HBOT, which augments oxygen diffusion without restoring arterial patency, PGE1 promotes vasodilation, enhances microvascular perfusion, inhibits platelet aggregation, and may exert cytoprotective effects on ischemic inner retinal tissue. Preliminary clinical studies, including early systemic PGE1 monotherapy reported by Takai et al. in 2013 and later observational cohorts, suggest that PGE1 may be particularly relevant for patients who present beyond the thrombolysis window, have contraindications to IVT, or reside in regions without HBOT access [44]. Beyond its hemodynamic benefits, PGE1 appears to attenuate oxidative stress, preserve mitochondrial function, and stabilize endothelial barrier integrity, thereby limiting leukocyte plugging and neuronal apoptosis. Collectively, these mechanisms position PGE1 as a mechanistically distinct complement or alternative to existing modalities and support growing interest in multimodal, pathophysiology-informed treatment algorithms for CRAO.

### 4.2. Health-System Logistics and Access Pathways

IVT can often be delivered rapidly under existing stroke protocols when CRAO is recognized early and when contraindications are absent. HBOT requires proximity to and coordination with a hyperbaric facility, which can introduce delay but may offer a therapeutic option for patients outside the thrombolysis window or with IVT contraindications. Developing local ‘CRAO codes’ that integrate ophthalmology, neurology, emergency medicine, and hyperbaric services may reduce time-to-treatment and standardize care [8,13,20,22,40].

## 5. Conclusions

Given the observational nature of the available evidence and the inherent heterogeneity across CRAO presentations, the pooled estimates generated in this review should not be interpreted as justification for routine use of HBOT or IVT in all patients. Treatment decisions must remain individualized, incorporating embolus characteristics, time from onset, contraindications, and local resource availability. Future research should prioritize well-designed comparative studies that incorporate emerging pharmacologic strategies such as PGE1 and evaluate therapies within pathophysiology-based treatment frameworks rather than relying on a binary comparison of HBOT versus IVT, so that patient selection and mechanistic suitability are appropriately integrated into therapeutic decision-making.

Cautionary statement: Within the constraints of heterogeneous observational evidence and indirect comparisons, both modalities are associated with visual improvement; however, we cannot infer comparative effectiveness. Safety spectra differ: IVT carries rare but potentially life-threatening hemorrhagic complications, whereas HBOT complications are predominantly quality-of-life threatening (e.g., barotrauma or anxiety). Treatment selection should consider onset-to-treatment time, local access, and patient-specific risk.

Both HBOT and IVT are associated with improved BCVA in CRAO. Differences in adverse event spectra and greater heterogeneity in IVT underscore the need for randomized, adequately powered trials and coordinated care pathways that account for onset-to-treatment logistics and resource availability. Until such data are available, treatment choice should consider local access, time from symptom onset, and patient-specific risk.

### 5.1. Pragmatic Clinical Pathway

A pragmatic approach is to triage suspected CRAO urgently using stroke protocols: confirm diagnosis clinically and with appropriate imaging; if within 4.5 h and no contraindications, consider IVT in collaboration with stroke services; in patients beyond the IVT window or with contraindications, consider expedited HBOT when available. Both pathways should be paired with secondary prevention and vascular risk work-up. Local resource availability and patient-specific risk should guide final decisions [8,12,13,14,15].

### 5.2. Standardized Outcome and Reporting Checklist

To facilitate comparability across future studies, we recommend standardized reporting of the following: (i) baseline BCVA and ischemic duration (onset-to-door, door-to-treatment); (ii) embolus characterization where feasible (e.g., retrobulbar spot sign); (iii) detailed HBOT protocol (ATA, minutes, number of dives) or IVT regimen (agent, dose, timing); (iv) adverse events using harmonized definitions; and (v) patient-centered outcomes beyond BCVA (e.g., reading vision, driving eligibility) [16,26].

### 5.3. Limitations and Future Directions

The evidence base remains observational, with potential for selection bias, confounding by indication, and center-level effects. IVT estimates were heterogeneous, complicating predictions for individual patients. Randomized, adequately powered trials comparing HBOT and IVT are needed, ideally with standardized eligibility criteria, imaging-based embolus characterization, and core outcome sets. Implementation science and health-economic evaluations are likewise needed to optimize care pathways across diverse health systems [15,16,22,26].

## Figures and Tables

**Table 1:** Study characteristics. N/A indicates data not reported in the original study.

Study ID	Treatment	Study Type	Sample Size (N)	Age Mean ± SD or Median (Range)	Sex (% Male)
Yang (2025) [40]	HBOT	Retrospective	39	60 (50–77)	53.8
Akai (2024) [17]	HBOT	Retrospective	11	70 (44–87)	50
Lopes (2019) [36]	HBOT	Retrospective	9	N/A	N/A
Masters (2019) [37]	HBOT	Retrospective	39	70 (30–93)	59
Lee (2024) [20]	HBOT	Retrospective	29	65.69 ± 15.22	58.6
Kim (2025) [33]	HBOT	Retrospective	41	74.3	53.7
Maldonado (2024) [21]	HBOT	Retrospective	114	68.9 ± 13.0	67.5
Chiabo (2024) [29]	HBOT	Prospective	31	68.3 (15–93)	38.7
Williamson (2023) [39]	HBOT	Retrospective	17	67.4 ± 14.6	64.7
Di Vincenzo (2022) [30]	HBOT	Retrospective	15	70.1 (16–89)	57.1
Rozenberg (2022) [22]	HBOT	Retrospective	121	69 ± 12	66.9
Lifson (2021) [35]	HBOT	Retrospective	15	68.7 ± 11.0	53.3
Coelho (2018) [18]	HBOT	Retrospective	14	68 (41–92)	57.1
Gupta (2019) [19]	HBOT	Prospective	62	65.3 ± 11.8	71
Jubran (2025) [25]	IVT	Retrospective	55	72	69.1
Stretz (2024) [28]	IVT	Retrospective	7	N/A	N/A
Kozner (2023) [34]	IVT	Retrospective	16	67.1 ± 13	81.3
Raber (2023) [38]	IVT	Retrospective	16	68.7 ± 14	56.3
Mac Grory (2020) [15]	IVT	Prospective	16	73.12 ± 11.9	62.5
Schultheiss (2018) [27]	IVT	Prospective	20	72.8 ± 10.9	50
Chen (2011) [24]	IVT	Prospective	8	73 ± 8	75
Alhayek (2024) [23]	IVT	Retrospective	13	68	61.5
Gilbert (2024) [31]	IVT	Retrospective	35	74 (41–93)	60
Hattenbach (2008) [32]	IVT	Retrospective	28	63.3 (30–85)	67.9
Nedelmann (2015) [26]	IVT	Prospective	11	N/A	N/A

## Data Availability

All data supporting this study are publicly available from the original publications included in the meta-analysis. References to these studies are provided in the manuscript.

## Supplementary Materials

The following supporting information can be downloaded at: https://www.mdpi.com/article/10.3390/jcm15072628/s1, Table S1. Search Strategy; Table S2. Rate of BCVA Improvement; Table S3. Onset-to-Treatment Time; Table S4. Adverse Events; Table S5. Newcastle-Ottawa scale adapted for observational cohort studies; Zero star: the item is not registered in the article; Very good studies: 9 to 10 points; Good studies: 7–8 points; Satisfactory studies: 5–6 points; Unsatisfactory studies: 0 to 4 points; Cohort studies: A study can receive a maximum of one star for each numbered item in the Selection and Result categories. File S1. PRISMA Checklist HBOT IVT [45].
