# Supplementary material for: Hyperbaric Oxygen Therapy Versus Intravenous Thrombolysis in the Treatment of Central Retinal Artery Occlusion: A Systematic Review and Meta-Analysis
Source: J Clin Med. 2026 Mar 30;15(7):2628. doi: 10.3390/jcm15072628 (PMC13073387; doi:10.3390/jcm15072628)
Supplement: Supplementary file 1 [file jcm-15-02628-s001.zip › jcm-4173421 - HBOT IVT Supplementary Tables.pdf]

Supplementary Table S1. Search Strategy

Date initiated		05/27/2025
Database results	PubMed (n = 481)	(“central retinal artery occlusion" OR "retinal artery occlusion" OR CRAO) AND ("hyperbaric oxygen" OR HBOT OR "oxygen therapy" OR "thrombolysis" OR thrombolytic OR "tPA" OR "tissue plasminogen activator" OR alteplase)
	Cochrane Library [Reviews excluded by filter] (n = 36)	("central retinal artery occlusion" OR "CRAO" OR "retinal artery occlusion") AND ("hyperbaric oxygen" OR HBOT OR "oxygen therapy" OR "thrombolysis" OR "tissue plasminogen activator" OR tPA OR alteplase OR thrombolytic)
	Web of Science [Reviews excluded by filter] (n = 296)	ALL=("central retinal artery occlusion" OR "CRAO" OR "retinal artery occlusion") AND ALL=("hyperbaric oxygen" OR "HBOT" OR "oxygen therapy" OR "thrombolysis" OR "tissue plasminogen activator" OR "tPA" OR "alteplase" OR "thrombolytic")
	Virtual Health Library (VHL) (n = 271)	(("central retinal artery occlusion" OR CRAO OR "retinal artery occlusion")) AND ("hyperbaric oxygen" OR "HBOT" OR "oxygen therapy" OR "thrombolysis" OR "tissue plasminogen activator" OR "tPA" OR "alteplase" OR "thrombolytic")
Total before duplicates		1084
Duplicates		525
Total after duplicate removal		559

Supplementary Table S2. Rate of BCVA Improvement

Study ID	Treatment	% Improved	Criteria Used	P-Value
Yang (2025) [10]	HBOT	51.28%	Decrease in logMAR ≥ 0.3	P = 0.021
Lopes (2019) [32]]	HBOT	55.56%	Decrease in logMAR ≥ 0.3	N/A
Masters (2019) [12]	HBOT	71.79%	None[1]	N/A
Lee (2024) [13]	HBOT	44.83%	Decrease in logMAR ≥ 0.3	P < 0.03
Kim (2025) [14]	HBOT	48.78%	Decrease in logMAR ≥ 0.3	P = 0.08
Maldonado (2024) [15]	HBOT	45.61%	Decrease in logMAR ≥ 0.3	N/A
Chiabo (2024) [16]	HBOT	51.61%	Decrease in logMAR ≥ 0.3	N/A
Williamson (2023) [17]	HBOT	47.06%	None[1]	N/A
Di Vincenzo (2022) [18]	HBOT	66.67%	Decrease in logMAR ≥ 0.3	N/A
Rozenberg (2022) [19]	HBOT	19.83%	Achieved BCVA of 20/200 or better	P = 0.523
Lifson (2021) [20]	HBOT	26.67%	Vision improved from no light perception (NLP), light perception (LP), hand motion (HM), or count fingers (CF) to a BCVA of 20/50 or better	N/A
Coelho (2018) [33]	HBOT	71.43%	Decrease in logMAR ≥ 0.3	N/A
Gupta (2019) [34]	HBOT	64.52%	Decrease in logMAR ≥ 0.3	N/A
Kozner (2023) [23]	IVT	43.75%	None[1]	N/A
Raber (2023) [24]	IVT	18.75%	None[1]	N/A
Mac Grory (2020) [25]	IVT	56.25%	Decrease in logMAR ≥ 0.3	N/A
Schultheiss (2018) [26]	IVT	30%	None[1]	N/A
Chen (2011) [27]	IVT	25%	Decrease in logMAR ≥ 0.3	N/A
Gilbert (2024) [29]	IVT	54.29%	Decrease in logMAR ≥ 0.3 and/or from a BCVA of 20/200 or worse to 20/100 or better	P < 0.05
Hattenbach (2008) [30]	IVT	32.14%	Decrease in logMAR ≥ 0.3	P = 0.004
Nedelmann (2015) [31]	IVT	72.73%	None[1]	N/A

Note: A decrease in logMAR ≥ 0.3 is equivalent to a gain of ≥ 3 lines on the Snellen visual acuity chart
N/A: not available
[*]No criteria in this study. Any improvement is included

Supplementary Table S3. Onset-to-Treatment Time

Study ID	Treatment	Time Mean ± SD (hours)	Time Median (IQR/Range) (hours)
Yang (2025) [10]	HBOT	N/A	144 (72, 264)
Akai (2024) [11]	HBOT	9.64 ± 6.14	N/A
Lopes (2019) [32]	HBOT	N/A	9 (2-20)
Lee (2024) [13]	HBOT	86.69 ± 135.76	N/A
Kim (2025) [14]	HBOT	11.2	9.5 (6.5, 14.0)
Maldonado (2024) [15]	HBOT	12.2 ± 6	N/A
Williamson (2023) [17]	HBOT	12.4 ± 4.9	(3-24)
Rozenberg (2022) [19]	HBOT	9.1 ± 5	N/A
Coelho (2018) [33]	HBOT	N/A	8 (2-24)
Gupta (2019) [34]	HBOT	7.3 ± 4.1	N/A
Jubran (2025) [21]	IVT	N/A	3.75
Kozner (2023) [23]	IVT	N/A	5.0 (3.8, 7.9)
Raber (2023) [24]	IVT	N/A	3.00 (2.50, 4.17)
Mac Grory (2020) [25]	IVT	< 4.5	N/A
Schultheiss (2018) [26]	IVT	3.058 ± 1.033	N/A
Chen (2011) [27]	IVT	14.4 ± 6.5	N/A
Alhayek (2024) [28]	IVT	2.67	2.63 (1.12-4.33)
Gilbert (2024) [29]	IVT	2.65	2.57 (0.30-4.37)
Hattenbach (2008) [30]	IVT	6.46	(1.5-12.0)
Nedelmann (2015) [31]	IVT	4.96 ± 2.59	4.25 (1.75-10.5)

N/A: not available

Supplementary Table S4. Adverse Events

Study ID	Treatment	Adverse Event	Frequency (n/N)
Yang (2025) [10]	HBOT	None	0/39
Akai (2024) [11]	HBOT	Worsening of loss of hearing	1/11
		Premature stop	2/11
Lopes (2019) [32]	HBOT	None	0/9
Masters (2019) [12]	HBOT	Need for incisional myringotomies	9/39
Lee (2024) [13]	HBOT	None	0/29
Kim (2025) [14]	HBOT	Neovascularization	10/41
		Ear barotrauma	1/41
Maldonado (2024) [15]	HBOT	Ear barotrauma	7/114
Chiabo (2024) [16]	HBOT	Ear barotrauma	6/31
		Arterial hypertension	9/31
Williamson (2023) [17]	HBOT	Hemotympanum	3/17
		Anxiety	2/17
Di Vincenzo (2022) [18]	HBOT	Hypertension	7/15
		Ear barotrauma	6/15
Rozenberg (2022) [19]	HBOT	Ear barotrauma	2/121
		Seizures and epistaxis	2/121
Lifson (2021) [20]	HBOT	Ocular neovascularization	3/15
Coelho (2018) [33]	HBOT	Ear barotrauma	1/14
Gupta (2019) [34]	HBOT	Otalgia	2/62
Jubran (2025) [21]	IVT	Minor systemic hemorrhage	1/55
		Recurrent RAO	3/55
		Stroke	1/55
Stretz (2024) [22]	IVT	None	0/7
Kozner (2023) [23]	IVT	Ocular ischemic complications	6/16
Raber (2022) [24]	IVT	None	0/16
Mac Grory (2020) [25]	IVT	Intracerebral hemorrhage	1/16
Schultheiss (2018) [26]	IVT	Angioedema	1/20
		Bleeding and hemorrhage from abdominal aortic aneurysm	1/20
		Recurrent CRAO	1/20
		Silent cerebral infarct	3/20
Chen (2011) [27]	IVT	Intracranial hemorrhage	1/8
		Retina neovascularization	1/8
Alhayek (2024) [28]	IVT	Intracerebral hemorrhage	1/13
Gilbert (2024) [29]	IVT	Intracerebral hemorrhage	2/35
Hattenbach (2008) [30]	IVT	None	0/28
Nedelmann (2015) [31]	IVT	None	0/11

Supplementary Table S5. Newcastle-Ottawa scale adapted for observational cohort studies; Zero star: the item is not registered in the article; Very good studies: 9 to 10 points; Good studies: 7-8 points; Satisfactory studies: 5-6 points; Unsatisfactory studies: 0 to 4 points; Cohort studies: A study can receive a maximum of one star for each numbered item in the Selection and Result categories.

	**Quality Assessment Criteria**								
**Author (Year)**	**Selection**				**Comparability**	**Outcome**			**Total**
	**Representativeness of exposed cohort**	**Representativeness of non-exposed cohort**	**Ascertainment of exposure**	**Outcome of Interest Not Present at Start of Study**	**Comparability of cohorts**	**Assessment of outcome**	**Adequate duration of follow-up**	**Adequate follow-up**	
Yang et al. (2025)	*	*	*	*			*	*	6
Akai et al. (2024)	*	*	*	*			*	*	6
Lopes et al. (2019)	*		*	*			*	*	5
Masters et al. (2019)	*		*	*			*	*	5
Lee et al. (2024)	*	*	*	*	**		*	*	8
Kim et al. (2025)	*		*	*			*	*	5
Maldonado et al. (2024)	*		*	*			*	*	5
Chiabo et al. (2024)	*		*	*			*	*	5
Williamson et al. (2023)	*		*	*			*	*	5
Di Vincenzo et al. (2022)	*		*	*			*	*	5
Rozenberg et al. (2022)	*	*	*	*	**		*	*	8
Lifson et al. (2021)	*	*	*	*	*		*	*	7
Coelho et al. (2018)	*		*	*			*	*	5
Gupta (2019)	*		*	*			*	*	5
Jubran et al. (2025)	*	*	*	*	*		*	*	7
Stretz et al. (2024)	*		*	*	*		*	*	6
Kozner et al. (2023)	*	*	*	*	**		*	*	8
Raber et al. (2022)	*	*	*	*	**		*	*	8
Mac Grory et al. (2020)	*	*	*	*	*		*	*	7

Schultheiss et al. (2018)	*	*	*	*	*		*	*	7
Chen et al. (2011)	*	*	*	*	*		*	*	7
Alhayek et al. (2024)	*	*	*	*	**		*	*	8
Gilbert et al. (2024)	*	*	*	*	*		*	*	7
Hattenbach et al. (2008)	*	*	*	*	*		*	*	7
Nedelmann et al. (2015)	*		*	*			*	*	5

** indicates two stars awarded for comparability.
